# Exploring the Financial, Psychological, and Operative Factors Associated With Implant Removal Satisfaction in Breast Implant Illness Patients

**DOI:** 10.1093/asjof/ojad027.015

**Published:** 2023-04-14

**Authors:** Corey Bascone, Javier Couto, J Reed McGraw, Reena Sulkar, Robyn Broach, Paris Butler, Stephen Kovach

**Affiliations:** University of Pennsylvania, Philadelphia, PA; University of Pennsylvania, Philadelphia, PA; University of Pennsylvania, Philadelphia, PA; University of Pennsylvania, Philadelphia, PA; University of Pennsylvania, Philadelphia, PA; Yale University, New Haven, CT; University of Pennsylvania, Philadelphia, PA

## Abstract

**Goals/Purpose:**

Breast implant illness (BII) is a poorly understood heterogeneous disorder treated with implant removal; however, patient-reported symptoms and outcomes following treatment remain unclear.

**Methods/Technique:**

A retrospective review of patients undergoing bilateral breast implant removal related to BII by two surgeons between 2018-2022 was performed. Patients were surveyed with the BREAST-Q augmentation model with the American Society of Plastic Surgeons (ASPS) BII survey extension. Outcomes were analyzed using a multivariable logistic regression adjusting for patient-associated factors.

**Results/Complications:**

Forty-seven patients were surveyed with a response rate of 51% (n=24). Of the 20 patients completing the survey, the majority were Caucasian (85%), with 45% (n=9) having a documented history of psychiatric illness. Six (30%) had capsular contracture and four (20%) had documented implant rupture. The majority of implant removals (n=12, 60%) were not covered by insurance. Fourteen (70%) had a net improvement in their symptoms after implant removal—most commonly improving chest discomfort, muscle pain, fever, and headaches. Self-pay was predictive of increased breast satisfaction scores (p=0.009), but had no impact on symptomatic improvement. Reduced time to implant removal was predictive of fewer residual symptoms (p=0.032). Capsular contracture was predictive of reduced psychosocial, sexual, and breast satisfaction scores (p=0.015). Psychiatric illness had no significant impact on outcomes.

**Conclusion:**

In the setting of suspected or diagnosed BII, reduced time to implant removal may decrease the risk of residual symptoms and improve overall patient satisfaction. In patients with capsular contracture, preoperative counseling should emphasize the fact that implant removal may improve physical symptoms only.

